# Phenomenological Evaluation of an Undergraduate Clinical Needs Finding Skills Through a Virtual Reality Clinical Immersion Platform

**DOI:** 10.1007/s43683-024-00139-5

**Published:** 2024-03-05

**Authors:** Christine E. King, Dalton Salvo

**Affiliations:** 1 Department of Biomedical Engineering, University of California Irvine, Irvine, CA, USA; 2 Department of English, University of California Irvine, Irvine, CA, USA

**Keywords:** Biomedical engineering education, Clinical immersion, Design ideation, Unmet clinical needs finding, Virtual reality

## Abstract

Clinical immersion programs have been widely used in higher education, particularly in biomedical engineering (BME) programs, to allow students to identify and evaluate the unmet clinical needs. However, due to limited space and extensive safety protocols required to enter hospitals, access for undergraduate students to shadow physicians is limited. BME students need to be trained to identify and evaluate unmet clinical needs through observation and experience to remain competitive in the medical device landscape. To be able to allow students to immerse themselves into clinical environments remotely and be able to visualize the full spectrum of healthcare workers and equipment that support a procedure beyond what is available through online video records, we have developed a virtual reality (VR) clinical immersion platform. The applicability and overall student satisfaction of using VR learning environments was investigated through a pilot undergraduate BME unmet clinical needs finding course in the spring of 2022. Pre- and post-course survey analyses revealed that the VR clinical immersion experiences did greatly increase immersion within a medical procedure, and students felt sensations of presence and embodiment. Given the results of this study, VR learning environments can provide a means for students to perform unmet clinical needs through virtual clinical immersion. However, these technologies to support environments should be readily accessible within the classroom.

## Introduction

In the realm of biomedical engineering (BME) curricula, the discovery of unmet clinical needs and the adoption of clinical immersion programs have become more widespread [1-3]. Regrettably, studies have found that only 48% of applicants to clinical immersion courses and 24% of applicants to immersion programs are able to participate annually [4]. The current situation leaves a majority of students unable to participate in any clinical immersion course or program. Given increasing cohort sizes and numbers of BME undergraduate programs [5], this suggests an increase in applicants to such clinical immersion experiences. As these programs continue to have substantially more applicants than can be served, programs such as those described in Guilford et al. [4] and Moravec et al. [6] illustrate that an inequitable situation exists wherein a majority of students will be unable to participate in any clinical immersion experience despite clear demand for such.

To allow BME students to fully engage with clinical settings, they must cultivate an understanding of the daily operations and necessities of the customers and consumers who will rely on their engineering solutions [7]. A lack of insight into how medical professionals employ these tools in their routine for diagnosis and treatment puts our students at a disadvantage. Though not an inevitability, a pervasive lack of human-centered design knowledge and understanding of how such devices are used in practice has the potential to hinder human-centered medical device innovation [8]. To stay ahead in the medical device domain, it’s imperative for BME undergraduates to be well-versed in spotting and addressing unseen clinical needs via direct observation and hands-on experiences [9]. Given the hurdles in accessibility and the recent pivot toward remote instruction, some educational bodies have turned to digital video sessions to achieve some degree of clinical immersion experience [10, 11]. Yet, these video resources don’t equate to the depth of immersion as in-person clinical environment exposure, neither do they present a comprehensive view of the myriad health professionals and apparatus involved in surgical processes. Thus, the onus is on educators to integrate technological tools that not only meet these academic aspirations but also amplify the students’ learning experience by focusing on enriching remote pedagogical materials and apparatuses.

The use of virtual reality (VR) technology has become increasingly popular in medical education and other educational settings within the last few years [12]. Such technologies use computationally rendered virtual places or virtualizations of physical locations that allow the user to dive into these virtual worlds and experience them through a first-person perspective [13]. These virtual environments (VEs) immerse the user in a simulated reality through the use of interactive devices such as Head Mounted Displays (HMDs), controllers, and data gloves. In the most common VR format the user dons a HMD through which they stereoscopically view a virtual or virtualized space and the HMD/controllers track the user’s movement, allowing them to “physically" navigate that virtual space and interact with any manipulable objects that may be present.

Early investigations indicate mixed outcomes from utilizing VR in educational settings [14]. Yet, newer studies contrasting VR with traditional video-based instruction reveal that VR learners display superior outcomes in terms of knowledge transfer and self-efficacy compared to their video-learning counterparts [15, 16]. This enhanced outcome can be attributed to VR’s capability to foster a heightened state of presence [17, 18]. By facilitating physical locomotion and interaction within the virtual space, VR technology aids in honing both hands-on procedural and declarative knowledge [19, 20]. The heightened sense of agency emerging from immersive VR interactions [21] has been linked to improving both memory and knowledge retention [22]. This amplified experiential sensation or embodiment correlates with an increase in the total content retained by students [23]. Such profound involvement within a dynamic virtual realm, where users hold a degree of autonomy, intensifies immersion and, therefore, presence. This enhanced sense of agency and presence can increase students’ understanding of the content as well as introduce and prepare them for various career paths in future [24].

Such student-centric pedagogical approaches encourage active learning by generating a greater felt sense of embodiment and agency through an integration of digital technologies and can increase levels of student engagement [25-28]. A prior study that compared 2D videos to an immersive and physically interactive 3D simulation demonstrated that students had increased levels of engagement, more positive attitudes toward the educational material, and the 3D environment had an overall greater pedagogical effectiveness [29]. While this study was conducted using a Mixed Reality simulation, it is likely the same would be true of a VR simulation given the underlying pedagogical principles remain the same. Consequently, in response to the aforementioned educational needs, the authors are crafting a VR-based clinical immersion curriculum for undergraduate BME students. This equips them to identify and evaluate unaddressed clinical needs before embarking on their yearlong senior entrepreneurial capstone course, referred to as the BioENGineering INnovation and Entrepreneurship (BioENGINE) program [30].

In this study, we investigate the practical application, potential pedagogical benefit, and overall student satisfaction of using VR learning environments to perform clinical immersion for unmet clinical needs finding among BME undergraduate students prior to entering the BioENGINE program. By assessing our students’ responses as to the educational efficacy of VR learning environments and whether they generate a greater degree of felt presence, we hope to discover if VR clinical immersion experiences are a viable and more effective alternative to traditional 2D videos. In the spring of 2022, the Biomedical Engineering Department at the University of California Irvine (UCI) introduced a unique clinical needs program available to all engineering departments. Drawing from insights provided by nursing and allied health experts, our material was designed to be universally accessible from any device, be it a phone, tablet, computer, or VR headset. With a blended online curriculum, encompassing VR immersive settings, video content, physician dialogues, educational modules, and collaborative classroom activities, we catered the learning modules to around 150 students given our current undergraduate program size. To assess and improve these learning modules prior to making the course a required course in the undergraduate BME curriculum, it was piloted among 22 students as an elective course. Our mission is to instruct students on identifying and evaluating unmet clinical needs that have the potential for commercialization, hone their teamwork competencies, and harness existing clinical and behavioral research instruments to enhance healthcare [31]. Effective clinical needs finding skills go beyond information retention and require the development of a critical awareness that can evaluate potential problems and create a solution to those problems. Given this, it must be noted that the aforementioned research results do not provide direct support that VR learning environments will be more effective in developing students' clinical needs finding skills versus 2D videos. It is unlikely that VR clinical immersion programs will be as pedagogically effective as in-person observation. However, we hypothesize that the first-person perspective allowed by VR, and the enhanced sense of presence and agency it generates, has the potential to be more beneficial to students’ development of such skills than 2D videos as it more closely simulates the experience of being physically present in the operating room. That said, and as we will discuss in greater length in our Discussions section, we were unable to objectively evaluate the pedagogical effectiveness of each version on equal grounds; rather, we focused on discovering our students’ opinions on both the 2D and 3D VR versions derived from their subjective experiences with the content.

## Methods

### Preparation Before the Experimental VR Course

Prior to our experimental course, we recorded our medical footage and constructed our virtual infrastructure. We filmed various medical procedures, but chose five operations in specific to evaluate our course and VR environments: Deep Brain Stimulation (DBS), Transcatheter Aortic Valve Replacement (TAVR), Spinal Deformation Surgery, Cataract Surgery, and a Corneal Transplant operation. These procedures were filmed using a 180-degree stereoscopic camera to capture the entirety of the operating room as well as recording footage from the primary physician’s point of view (POV) through a head mounted camera worn during the operation. The filming of these procedures was approved as exempt under UCI Institutional Review Board (IRB) (IRB Exempt No. 15531 for UCI Medical Center filming and 1184 for UCI School of Medicine and School of Nursing filming locations), and all physicians, patients, and staff provided informed consent prior to filming. Additionally, we interviewed the primary acting physicians of each procedure as well as some engineers and entrepreneurs involved in the development of the medical devices used to provide supplemental learning materials. Once collected, the videos of the respective procedure and all the supplementary materials were included in the VR experience for that procedure. For example, in the TAVR environment, we included interviews from cardiologist Dr. Pranav Patel, engineer and VP of R&D at Edwards Lifesciences Sean Chow, an animation of a TAVR procedure, an overview of Edwards Lifescience’s SAPIEN XT Transcathether valve that is used in this procedure, and the video footage of the operation itself. As described in “Unmet Clinical Needs Evaluation” section, we built a rudimentary hospital environment with various rooms. Given the limitations of the game design software used, we were unable to house multiple videos within the same space. To resolve this issue, each room serves as a launching point that will transfer the user to a new environment wherein they can review the learning materials located in that room.

Given our goal to maintain the highest level of accessibility, we created both a 2D traditional video version and 3D VR version of each experience deployed across a range of platforms and devices using the same footage described above. Our 2D experiences consisting of the operating room footage with the physician’s POV overlaid, and the physician and engineer interviews were uploaded to our YouTube channel (https://www.youtube.com/channel/UCAwi0hckuth-OGOAOIB8YEg) so that anyone with a digital device and internet connection can view them. We also created a 2D PC version using the Unreal Engine game development software (Unreal Engine, Cary, NC), which allowed us to embed all of our content within a single interactive and explorable virtual space similar to contemporary PC video games. For our 3D VR versions, we uploaded the operating room footage to YouTube so they could be viewed using a Google Cardboard VR headset (Google, Mountain View, CA) or Meta Quest 2 VR headsets (Meta, Menlo Park CA), which could be checked out from our library [32]. Additionally, we developed an immersive and interactive version using the Unreal Engine specifically for the Meta Quest 2 headsets, allowing us to include all the relevant materials for a specific operation within a single virtual environment. For both the 2D PC and interactive VR experiences, we created one version that relied on streaming videos from our AWS servers for the highest quality content, but also a downloadable version for those who do not have the necessary bandwidth to stream high-quality video content. By developing various versions of each experience and deploying our content across different platforms and devices, we ensured that any student with a smartphone, tablet, computer, or VR headset with internet access could view our educational content.

Our participants consisted of undergraduate BME students enrolled in a pilot elective course, BME 179: Biomedical Engineering Design: Addressing Unmet Clinical Needs. This class is an upper division elective BME course consisting primarily of juniors and seniors with the occasional freshman and sophomore. This course is designed to introduce undergraduates to the non-technical aspects of medical device development by performing unmet clinical needs identification and evaluation. The goal is to use the virtual experiences to help our undergraduates develop their clinical needs finding and screening skills by exposing them to actual medical procedures, and using these procedures to evaluate identified needs for potential commercialization and further development in their senior capstone program.

### Stages of the Experimental VR Course

At the start of the course, we explained the nature of the experiment to the students and then asked them to fill out an optional survey to establish demographics, and their usage of both VR and traditional video games prior to them starting the class. All students were provided with an Information Study Sheet made available on the course website (UCI IRB Exempt No. 904). Additionally, this first survey also asked students how they think VR could be either beneficial or an impedance to developing clinical needs finding skills and gauge their overall interest in using VR for educational purposes (to view Survey 1’s Questions, see Appendix B.1).

During the course, the students watched guided video interviews from physicians and online virtual reality immersive surgeries and procedures that displayed how physicians and support staff conduct themselves during such procedures. They then identified potential unmet clinical needs and issues that engineering principles of design can be applied to. Prior to this, they formed teams based upon their interests and active learning modules within the classroom that enhanced team cohesion and effective teamwork [33]. The students then performed gap analyses as well as virtually interviewed potential customers, strategic partners, and stakeholders in the value chain using online surveys to evaluate whether there was any potential commercialization for their proposed solution. In addition, to gain further insight from stakeholders, students were able to interview healthcare professionals through zoom interviews with physicians and staff from UCI Medical Center and Children’s Hospital of Orange County that have ongoing collaborations with the course instructor (to view a sample of one team’s interview questions and answers, see Appendix C). They also performed online surveys with patients through patient advocacy groups. By the end of the quarter, the students drafted a strategic plan to develop their product throughout the BioENGINE program [30]. The following sections high-light how the course provides hands-on learning, VR clinical immersion experiences, business model canvas development for students to be able to identify and evaluate an unmet need, and how to create a viable design plan for their senior capstone course [34], which they perform the following year in a 9-month mentored entrepreneurial design program [30]. These learning modules are conducted to achieve the following major course learning outcomes:

1. Identify medical, technical, and market needs in formulating biomedical engineering solutions to unmet clinical needs (EAC 7).
2. Employ the various steps in bringing a biomedical product from ideation to invention (EAC 2).
3. Identify the realistic constraints of a medical innovation product, with consideration of public health, safety and welfare, as well as global, cultural, social, environmental, and economic factors (EAC 2).
4. Articulate the impacts of the project in a global, economic, environmental, and societal context (EAC 4).
5. Communicate unmet clinical needs, concept generation, and concept screening of medical innovation and inventions to a wide range of audiences (EAC 3).

It can be noted that each course learning objective described above meets ABET’s Engineering Accreditation Commission (EAC) student learning outcomes for BME programs. In particular, course learning objective 1 meets EAC 7: “an ability to acquire and apply new knowledge as needed, using appropriate learning strategies,” while learning outcomes 2 and 3 meet EAC 2: “an ability to apply engineering design to produce solutions that meet specified needs with consideration of public health, safety, and welfare, as well as global, cultural, social, environmental, and economic factors.” Course learning outcome 4 meets EAC 4: “an ability to recognize ethical and professional responsibilities in engineering situations and make informed judgments, which must consider the impact of engineering solutions in global, economic, environmental, and societal contexts,” while course learning outcome 5 meets EAC 3: “an ability to communicate effectively with a range of audiences” [35].

#### Technical Skills Development Through Reverse Engineering

**Fig. 1:**
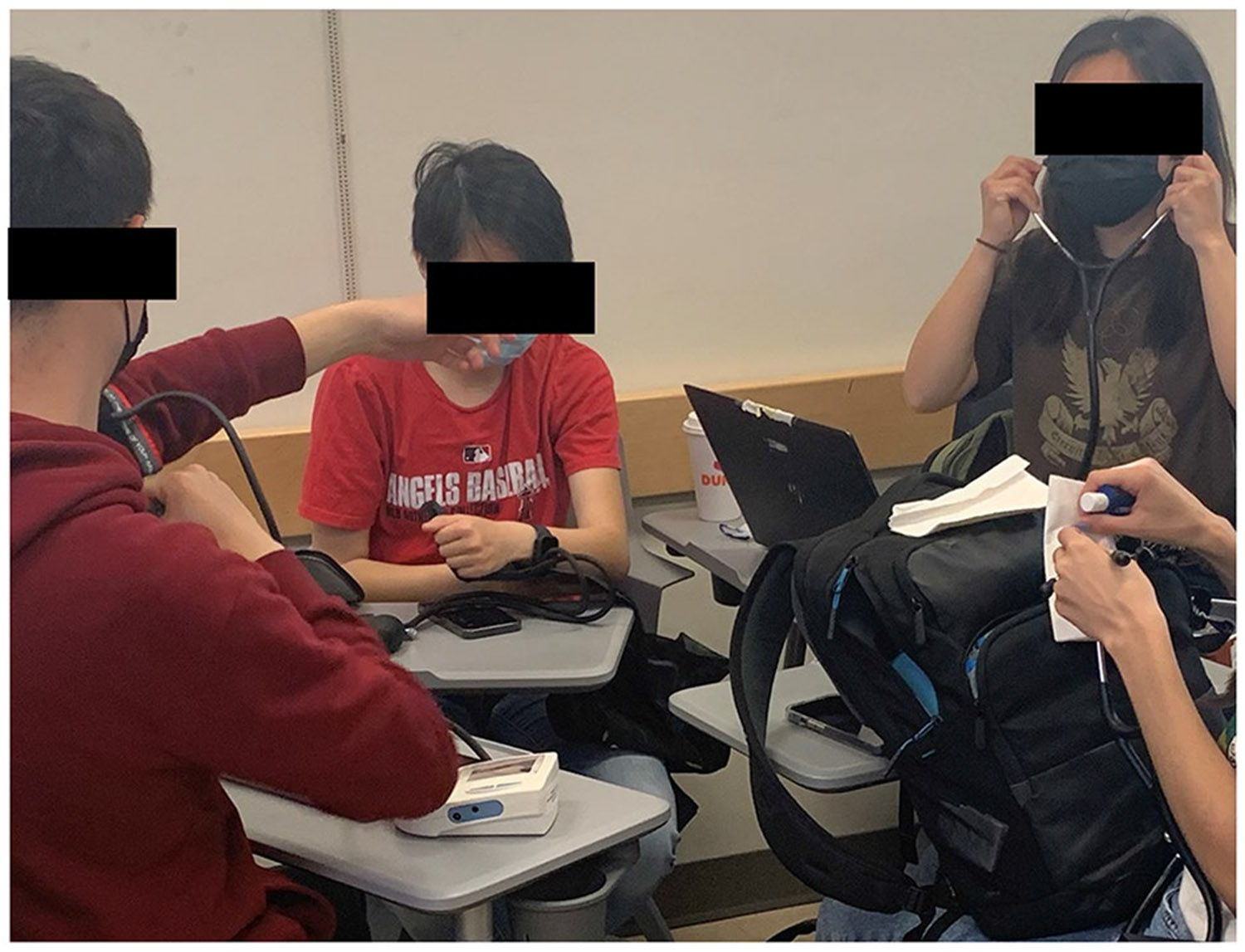
Image of students reverse engineering a BP cuff to learn how medical devices are designed

To understand how medical devices are designed, the second two weeks of the course focused on reverse engineering medical devices by the student teams to understand how devices work and are designed. This consisted of identifying an existing medical device in their team’s medical field of interest. Subsequently, they were tasked with grasping and elaborating on: 1) the context in which the device is employed (such as its application location, the target user, frequency of use, and duration), 2) its comprehensive functionality (e.g., dismantling and reassembling it), 3) intentional design choices (like choice of materials, safety features, electrical needs, and software specifications), 4) potential points of breakdown through a failure mode effects analysis, 5) user-friendliness (including aspects like comfort, recyclability, simplicity of implementation, and operation through human-centered design learning modules), and 6) potential design enhancements. Each group member presented insights based on their respective “specialty” regarding the design criteria of the current medical tool. As an illustration, a student representing the “electrical engineer” role detailed the circuitry and electrical evaluations of a blood pressure (BP) cuff required to take BP measurements from a pressure sensor, while the one emulating a “computer scientist” outlined the user interface, data gathering, and software processing components of the BP monitor (Fig. 1). After a regulatory controls lecture in the fourth week of the course, the students then performed an FDA strategy assignment to identify which standards would be required for their portion of the device. These exercises were mentored by the instructor and instructional team to guide them through probing questions based on how different types of engineering and science combine to develop a medical device, and to justify and expand upon the above tasks.

#### Virtual Unmet Clinical Needs Identification

**Fig. 2:**
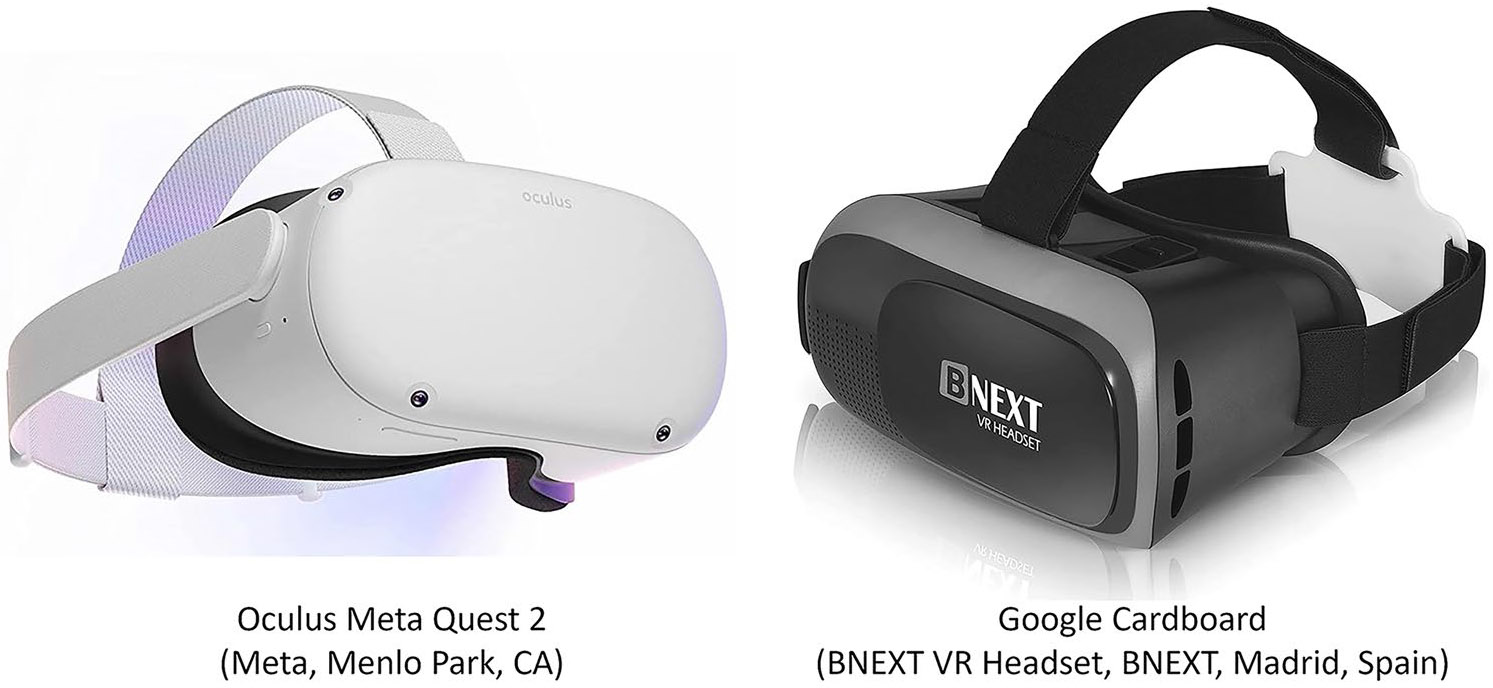
VR headsets that were provided to the students as part of their course materials. The BNEXT VR Headset was used as the Google Cardboard, which allowed students to watch videos in VR on YouTube using their cell phones

**Fig. 3:**
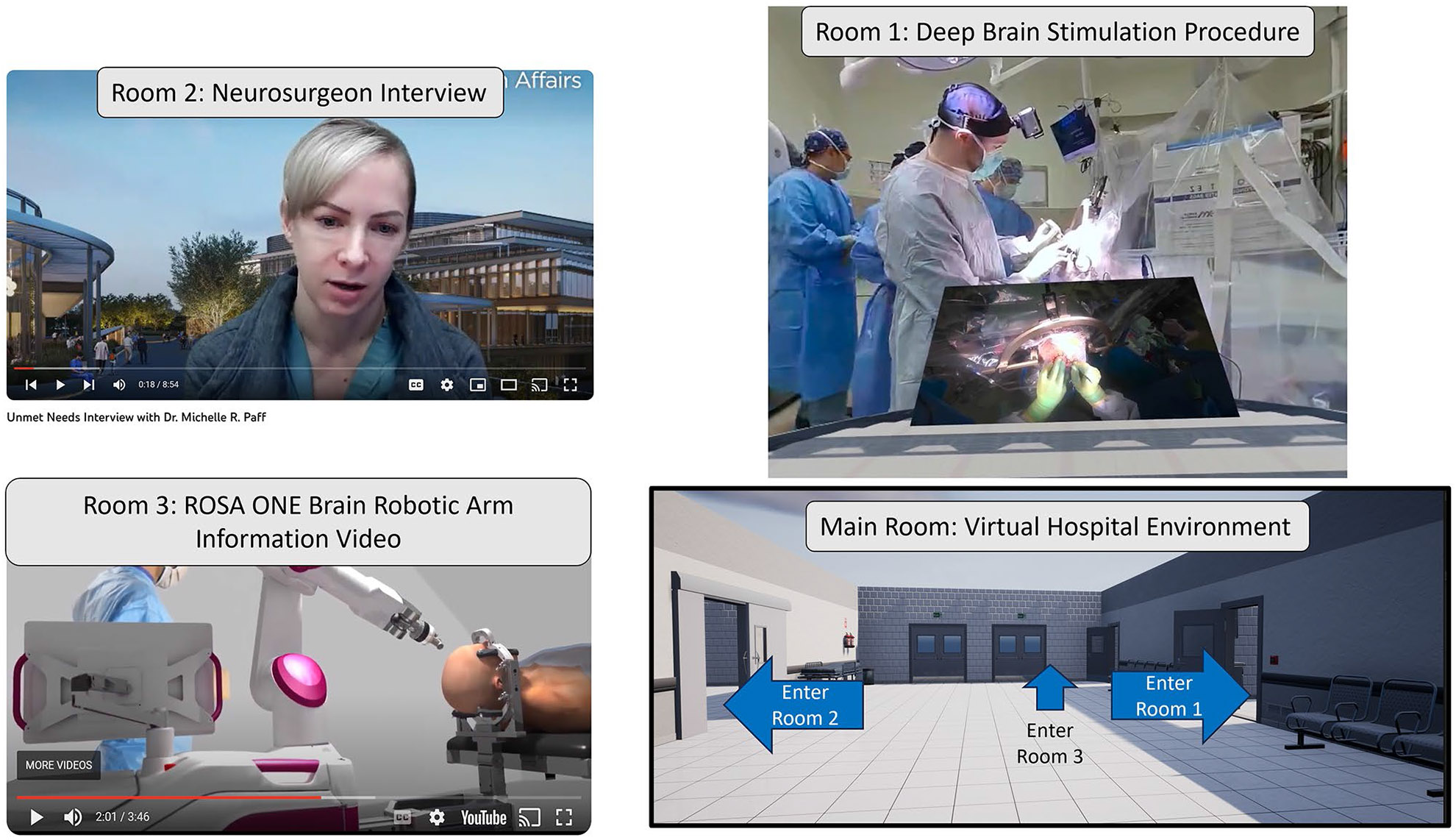
Views of the different “rooms” in the VR environment, including the main hospital environment where students can walk into: room 1) the VR medical procedure (e.g., a DBS procedure), room 2) an interview with the attending physician, and room 3) an informational video of the technology (ROSA ONE Brain, Zimmer Biomet, Warsaw IN)

After the reverse engineering exercises were performed, students used immersive virtual reality software that is compatible with all phones, computers, and virtual reality headsets, and google cardboard and Meta Quest 2 headsets (Fig. 2) were provided to them as part of their course materials and were available for loan at the school science library. These environments were designed to be able to allow students to perform clinical needs identification virtually by being able to go into a surgical suite to see the physician’s viewpoint as well as a 180-degree 3D view of the operating room. The students could also travel to other rooms to view the supplementary interviews and information (Fig. 3). Note that the full game-like environment presented in Fig. 3 was available in the 2D PC version and Meta Quest VR headset version, but was not possible to be used in the Google Cardboard’s version, as this required students to use their phones and YouTube videos. For the Google Cardboard version, students watched these videos through YouTube playlists, such as the interview playlist described below.

Alongside their virtual “tours” (Fig. 3, an example clip of the DBS procedure shown in the figure can be found here: https://www.youtube.com/shorts/ttDVAxnOQzU), they attended lectures and watched several online videos consisting of informational videos on the technologies used and guided interviews with physicians, therapists, nurses, staff, engineers, and entrepreneurs. These supplemental videos were used to understand the current needs of different environments that they encounter within the virtual experiences. The interviews were also provided via the following YouTube playlist: https://www.youtube.com/playlist?list=PLuCIsTqjwEZupB5MxySU6DPBpiPsaa42x. These interviews provided context behind each medical procedure and gave students an understanding of the history, current standards of care, and future research of the particular field. After having gone through the guided clinical needs finding exercises during the reverse engineering stage of this course, having selected their respective procedure, and having reviewed all the related content, each group performed homework assignments where they were asked to identify needs they can address as a biomedical engineer or other engineering discipline.

#### Unmet Clinical Needs Evaluation

After performing the learning modules using VR environments, videos, and supplemental material to identify medical devices that are currently used in clinical practice, student teams used their developed list of identified unmet clinical needs to evaluate which need is most viable to solve. This was founded on a series of tasks: 1) assessment of the market scenario, 2) approach to intellectual property and its challenges, 3) clinical approach and obstacles given current standards of care, 4) regulatory plan, and 5) reimbursement strategy. These concept screening and evaluation learning modules are based on *Stanford University’s Biodesign: The Process of Innovating Medical Technologies* textbook and program [36]. For assessment purposes, students kept an “innovation notebook” which documented their observations, conversations, and review sessions conducted in the VR setting and over the duration of the course. Instructors assessed these notebooks in terms of completeness, ensuring that more than three observations were performed, and that students had identified any problems with corresponding design criteria and constraints within each observation. They used the needs they had identified to determine which top three needs should be further evaluated in terms of their potential market, and what the best proposed solution would be to pursue throughout their senior capstone course.

### Quantitative and Qualitative Analysis After the Experimental Class

When experiencing a state of presence in a virtual environment, the person in question psychologically feels as if they are physically present in the virtual environment. Because presence is a perceptual phenomenon experienced by an individual, presence, then, is a property of that person. However, as Lombard and Ditton observe, presence “results from an interaction among formal and content characteristics of a medium and characteristics of a media user, and therefore it can and does vary across individuals and across time for the same individual” [37]. Thus, the sensations of presence and embodiment one feels in an immersive virtual environment are intensely subjective experiential phenomena. To effectively evaluate and refine our virtual experiences, we utilized a phenomenological approach, a frequent choice among VR researchers, during our user feedback collection and analysis. Phenomenology acknowledges and attempts to address how not only our body but also our environments, and the objects, entities, and stimuli of which they consist, play a role in shaping conscious thought and behavior when directly experienced. As such, it is commonly adopted by VR researchers who focus on the human experience of such technology [38].

Given that human awareness is influenced by mental, environmental, and physical sensations, a phenomenological approach guides us to focus on the engagements between users and the encompassing virtual environment. Subsequently, after the course completion, we requested our participants to fill out questionnaires to determine if the VR experiences increased their sense of presence and whether or not they found the VR versions more educationally beneficial than traditional 2D videos. We also had students list any ideas they may have for improving our educational content and the VR experiences as well as list any educational use cases they think VR could be effectively used to improve learning (to view Survey 2’s questions see Appendix B.2. Later, we initiated personal interviews with the volunteers among survey participants. In these interactions, our aim was to discern their real-time experiences in the virtual realm by seeking insights into their feelings of presence, embodiment, and their emotional responses to their immersion in a clinical space. We also asked students to follow-up on the survey question and provide any ideas they think could be used to improve our content and virtual experiences (to view the interview questions, see Appendix B.3).

## Results

### 3D VR Clinical Immersion Experiences Provide a More Immersive Learning Experience

Given the lack of physical access to operating room environments, one of our goals was to simulate, as closely as possible, the perspective of actually standing in the operating room during a procedure. Accordingly, we explored two primary questions. Firstly, does 3D VR increase a felt sense of presence? Secondly, do the students find our 3D VR clinical experience to be more or less educationally beneficial than traditional 2D video content? To make the content scalable and widely available across all higher education institutions, we focused on providing the same overall view of the procedures whether viewed in a traditional 2D video format or using our 3D VR experiences. As such, all versions presented students with a 180-degree view of the operating room along with a POV of the acting physician, allowing students to more fully grasp the spectrum of staff and equipment active during each respective procedure. Building our PC and VR experiences using Unreal Engine game design software [39] made it possible to locate all the respective content for each procedure (operation animations and footage, interviews, and medical device equipment information) within a single navigable space. Though to a minimal degree, these game-like experiences necessitated a level of conscious interactivity to view each element of the procedure by requiring navigation of the avatar to various locations as well as to access and manipulate the video content. Moreover, at a phenomenological level VR first works through sensory deprivation: depriving the user of the visual and auditory sense-data of the physical environment in which they are located; and secondly, by supplanting the visual and auditory sense-data of objective reality with that of the virtual world. The combination of a more realistic perspective made available by 3D VR technology, increased interactivity, and a forced narrowing of the students’ focus to solely the sense-data of the virtual experience created a more intensely immersive experience than traditional 2D videos.

### The Most and Least Educationally Beneficial Clinical Immersion Formats

**Fig. 4:**
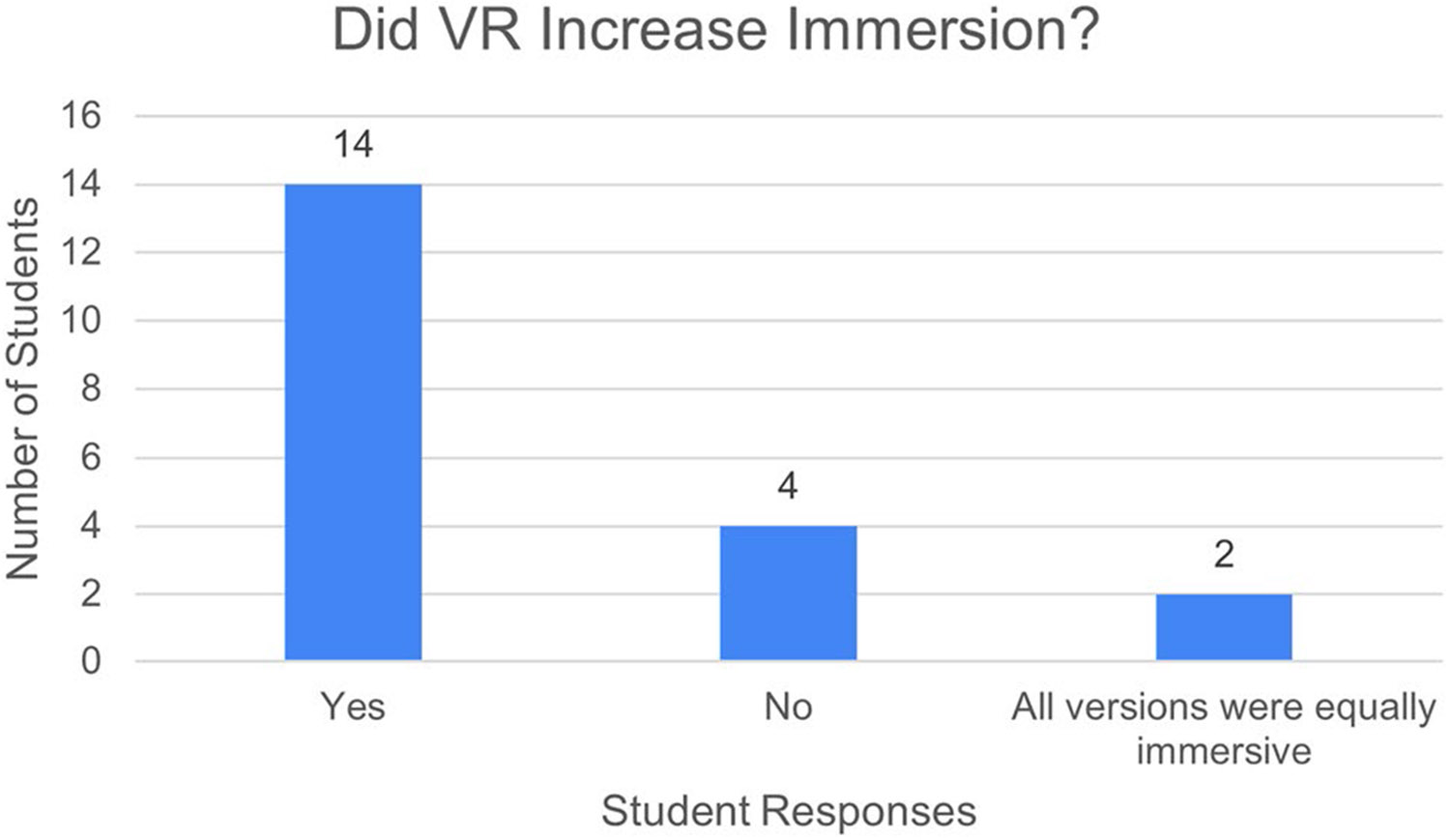
Responses to the post-course survey question “Did VR increase the felt sensations of immersion and presence?”

To investigate our hypothesis, the students completed a questionnaire at the end of the course and some participated in a brief interview. A breakdown of the student demographics for those who participated in the survey can be seen in Appendix Figs. 13, 14, 15, and 16. The goal of the survey was to identify whether or not the 3D VR clinical immersion experience elicits a greater degree of presence: the feeling that they are physically and/or psychologically present in the operating room. Students were asked if the 3D VR versions elicited a greater degree of presence or whether all the different versions were equal in this regard. 70% of students noted the VR version elicited a greater felt sense of presence, 10% felt that all were equal, and 20% opined that it did not increase their sense of presence (Fig. 4).

**Fig. 5:**
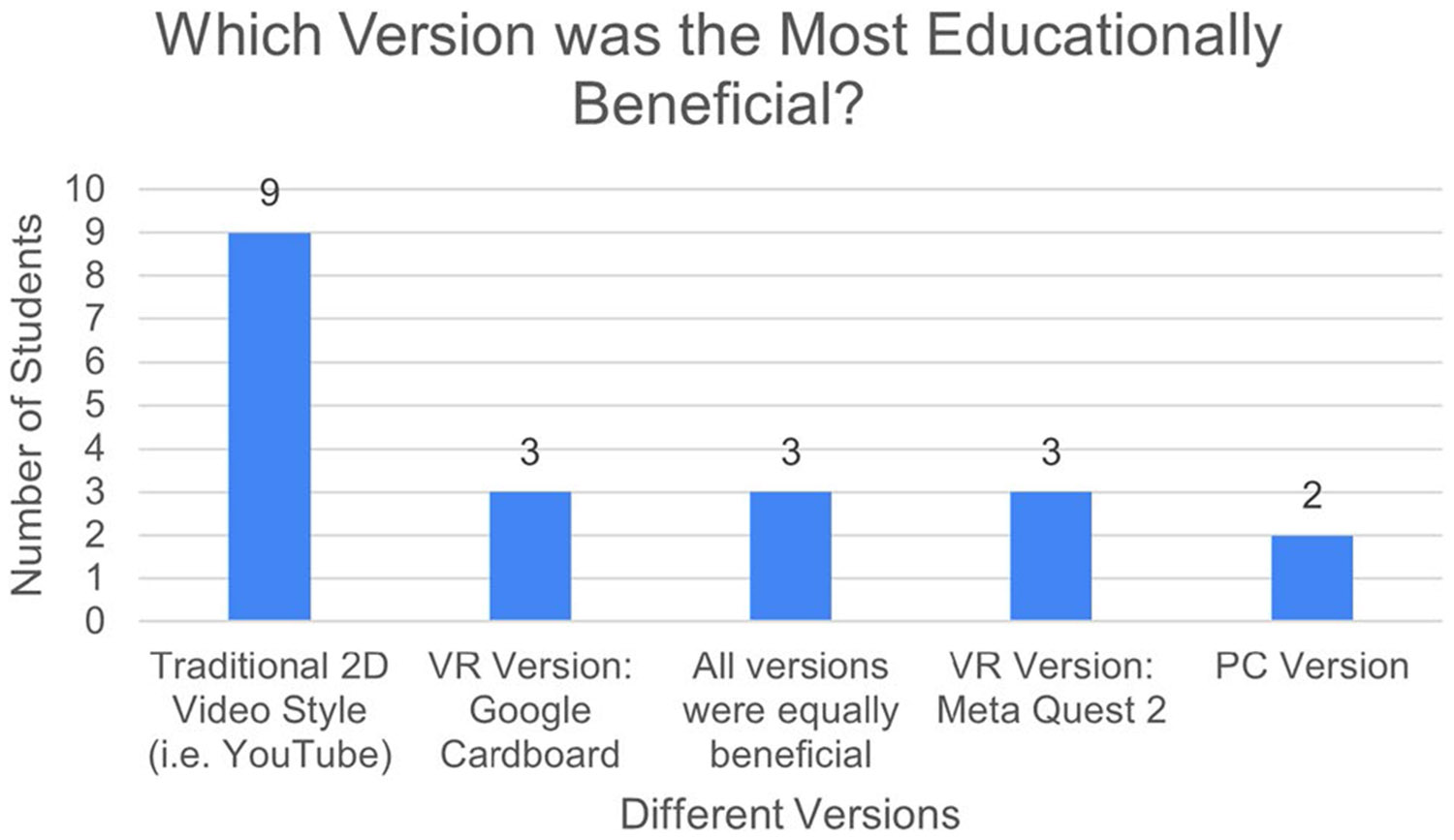
Responses to the post-course survey question "What was the most educationally beneficial version?"

Students were then asked to identify what they believed was the most and least educationally beneficial versions. There are numerous factors that can play a role in determining overall pedagogical efficacy of an educational experience including the method of content delivery, the visual fidelity or quality of the content, ease of access, interactivity, and physical comfort or discomfort among many others. Given how difficult it is to account for all such variations, we decided to take a more holistic approach by asking our students to evaluate the most and least beneficial version according to their subjective experience and to then explain what elements of that version made it better or worse than others. 40% of students felt the traditional 2D videos were the most beneficial, 20% chose the Google Cardboard, 20% chose the Meta Quest 2, 20% believed all were equally beneficial, and 10% selected the PC version (Fig. 5).

**Fig. 6:**
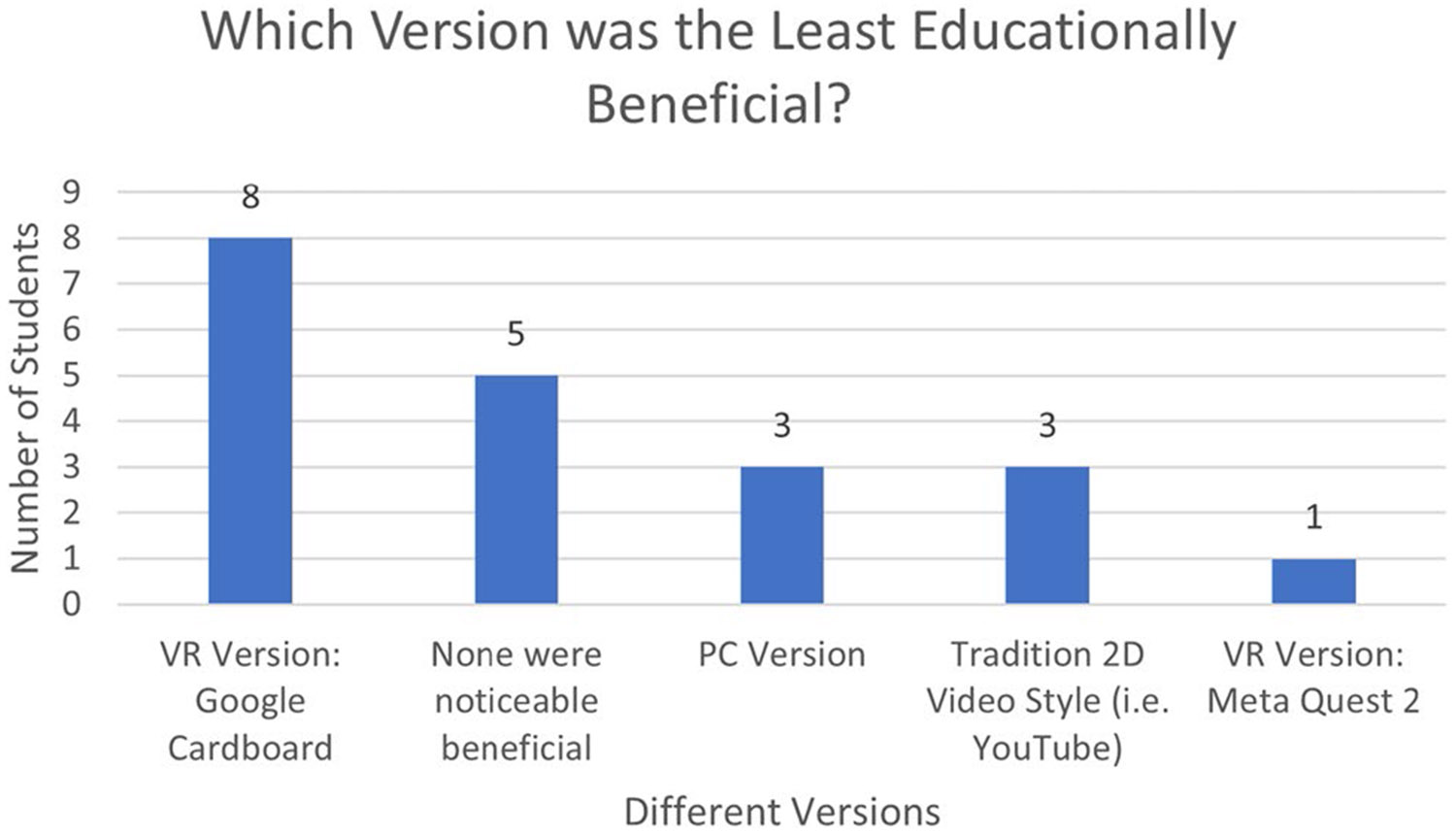
Responses to the post-course survey question "What was the least educationally beneficial version?"

As for the least educationally beneficial version, 40% of students claimed that the worst version was the Google Cardboard, 25% felt that none of the versions were noticeably beneficial to their learning, 15% chose the PC version, 15% chose the traditional 2D videos, and only 5% believed the Meta Quest version is the worst (Fig. 6).

**Fig. 7:**
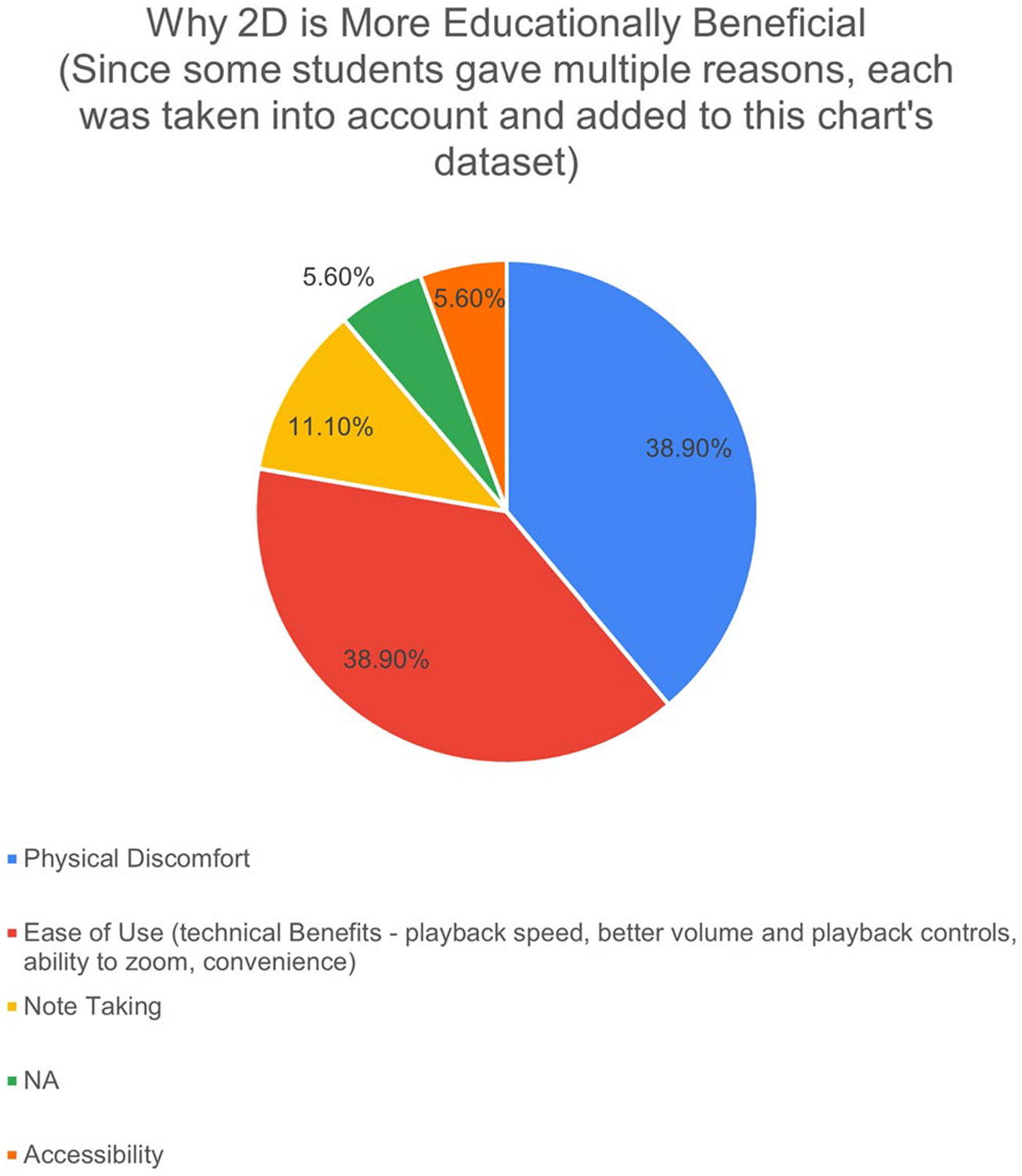
Percentages of the responses to the post-course survey question “Why 2D is More Educationally Beneficial?” (No. Responses = 20 students)

For those students who felt the 3D VR versions were the most beneficial, 66.6% stated that the increased sense of presence generated from the embodied first-person perspective offered by the HMD was what made it more beneficial, 26.7% claimed it was the increased ability to focus due to the removal of distractions when wearing the HMD, and 6.7% felt the affordability and accessibility of, specifically, the Google Cardboard are what made it the best (Appendix Fig. 8). The follow-up interviews corroborated that the two primary factors leading to their selection were the more realistic perspective offered by 3D VR and the sensory deprivation aspect helped them focus on the content by cutting out the various distractions one typically faces in any environment: computer and phone notifications, background sounds and movements, other people, etc. For those students who felt the 2D version is better, our interviews suggested the reason for this seems to lie in two primary factors: convenience/ease of use and physical discomfort. As one student described it, having to go to the library to check out the VR headset is “a hassle,” leading them to watch the YouTube videos on their phone or computer out of convenience. Furthermore, 44.5% claimed ease of use (convenience/accessibility, technical benefits such as variable playback speed, better volume and playback controls, and ability to zoom in to the footage) was the reason the 2D version is better. 38.9% stated that the physical discomfort (disorientation, weight of the headset for long periods of time, or the uncomfortability of wearing glasses with the headset) made the VR versions less beneficial. 11.1% felt the lack of being able to take notes while wearing the HMD made the VR versions worse, and 5.6% never tried the VR version at all, leading them to select the 2D videos as the best (Fig. 7).

### Prior Familiarity with VR and Traditional Video Games Effect on Choosing the Most Educationally Beneficial Format

One element we also wanted to explore was whether one’s familiarity with VR and video games in general prior to taking the course affected their choice on whether the VR version is better or worse educationally. While we expected that increased familiarity with VR and video games would lead those students to lean toward the VR versions, this was not the case. In fact, it seemed that the students with a higher level of familiarity with VR and/or video games were more likely to select the 2D video content as more educationally beneficial. Interviews indicated that this is due to the sheer lack of functionalities, visual fidelity, and overall polish of the VR versions as they do not meet the production standards of contemporary VR and traditional video games. For those students who felt the 2D version was more educationally beneficial, 66.7% had used VR at least once prior to taking the course, 11.1% had never tried VR, 11.1% used VR at least once every three months, and 11.1% used VR on a weekly basis (Appendix Fig. 9). As for traditional video game usage, 66.7% of students who picked the 2D version gamed on a weekly basis, 22.2% gamed at least once every three months, and 11.% gamed as a child or game very infrequently (Appendix Fig. 10). For those students who thought the VR versions were the most educationally beneficial, 66.7% had tried VR at least once prior to taking the course, and 33.3% had never tried VR before (Appendix Fig. 11). Regarding traditional video game usage, 33.3% gamed as a child or on a very infrequent basis, 33.3% gamed weekly, 22.2% gamed at least once a month, and 11.1% gamed at least once every three months (Appendix Fig. 12). Interestingly, given these survey results, there is a possible correlation between one’s familiarity with video games and their opinion as to the more educationally beneficial version of our content. While more research needs to be conducted for validation, superficially it appears as if the more a student plays video games, the more likely they are to select the 2D version as the most educationally beneficial.

## Discussion

Given the goal that our educational content should be open source and easily accessible for other BME programs to adopt, we had to make certain concessions in how the platform was offered that influenced our data in identifiable ways. For example, the cost-prohibitive nature of high-quality VR headsets like the Meta Quest 2 we used did not allow us to deploy their usage in the classroom itself, relying on the Google Cardboard HMDs instead. While we had several Meta Quest 2’s housed in the school’s library, not a single student went there to check one out. Because, as one student phrased it, “it was a hassle” to physically obtain a headset for loan from the library when they could just watch the YouTube videos on their phone or computer, most of our students never tried the Meta Quest 2 during the course. Out of those that did, almost all only viewed the YouTube videos rather than using the downloadable and interactive VR game-like versions. As some research has shown, convenience is a crucial criteria for students over and against other factors and students associated convenience with perceived learning and course satisfaction [40]. Additionally, with regard to information-seeking practices in both academic and everyday-life contexts, convenience is one of the primary criteria at work in making decisions throughout the information-seeking process to the point where, in some situations, quality or content will be readily sacrificed for the sake of convenience [41]. Our student interviews corroborated this, specifying convenient ease of access and quality of life features like being able to adjust playback speed in YouTube but not in VR as the primary factors leading to them either not checking out the VR headsets from the library or using the downloadable interactive VR experiences. In retrospect, our commitment to open access across a variety of devices and platforms apparently moved students to use the most convenient option available to them, making it impossible to properly evaluate the pedagogical efficacy of each version on equal grounds.

Another problematic element of our interactive VR learning environments is the lack of any real-time collaborative features. Students could only enter the VR environments by themselves and could not work with others in the same virtual environment. While we do intend to address this issue in future iterations, our VR learning environments will not be suitable for any kind of group work or team meetings until such functionality is added. Furthermore, the elements of the VR versions which students noted as detrimental beyond convenience, such as physical disorientation and discomfort, unwieldy controls, and lack of quality of life and collaborative features, were illuminating. It is likely that some of these issues would have been less relevant had the students used the high-quality Meta Quest 2 rather than the Google Cardboard, which is a relatively horrible experience by comparison. But this alone is not enough to suggest that our students would have selected the interactive VR versions as the most beneficial even if they had taken the time to check the Quest 2’s out of the library. So while it is indisputable that a majority of our students felt that VR increased their sense of presence and immersion, we cannot conclusively claim that interactive VR learning environments are more or less pedagogically efficacious compared to 2D videos at this point in time.

There also was an unexpected correlation between our students’ familiarity with traditional 2D gaming and VR usage and their overall opinion of our virtual learning environments. It seems as if the more familiar or often our students game or used VR prior to taking this course, the more likely they were to select the 2D version as the most educationally beneficial. Conversely, our students who were less familiar with gaming and VR prior to taking the course were more likely to select the VR version as being most beneficial. This was a completely unforeseen result. While some of our students did account for various elements such as the lack of collaborative functionality, we did not ask our students to address how and/or why their familiarity with gaming may have influenced their overall opinion of the effectiveness of our virtual learning environments. What follows, therefore, is mostly our own speculation.

One possible reason for this is the sheer novelty of experiencing VR or video game-like environments. In which case, as the students continue to use VR for educational purposes the novelty factor will degrade over time, lessening the students’ perceived pedagogical effectiveness of the technology. While this is most definitely one factor, we do not believe this to be the primary criteria in overall student dissatisfaction. Rather, the nature of our interactive VR and PC based learning environments fundamentally relies on game engine software and game based mechanics for movement and interactions within the virtual space. Thus, a comparison between our rudimentary environments and traditional consumer gaming experiences is inevitable. In such a light, the visual fidelity or quality of our learning environments, the amount of and types of interactivity made available, and the sheer lack of features found in traditional games like multiplayer functionality, voice chat, and customizable avatars is severely lacking. Without a fully fledged professional development team, it is simply not possible to produce virtual learning environments with the same quality and functionality as what is found in consumer gaming products. We believe this to be a crucial finding considering the pervasive use of video games and the general increase in the number of gamers worldwide. While additional research will be necessary to validate this, if this correlation turns out to hold true it suggests that game-like virtual learning environments will be less positively received by students until they begin to approach feature, if not quality, parity with user expectations of and experiences with consumer video games. We still believe virtual learning environments hold tremendous pedagogical potential for developing clinical needs finding skills when in-person clinical access is not an option and serve as a viable alternative to 2D videos. However, definitive success of interactive virtual learning environments will likely depend on massively increasing the quality and functionality of said environments as well making it more convenient and cohesively integrated into the day-to-day workflows of our students.

Another limitation of the study is with regard to how the VR content was filmed within the medical spaces. In particular, the medical procedures were filmed using a 180-degree stereoscopic camera to capture the entire operating room as well as the primary physician’s POV through a head mounted camera. Due to the locations of the surgical lighting structures and other ceiling mounted equipment within a surgical suite, the need to film across different rooms, and hospitals given limited funding for camera equipment, a full 360 view of the environment was not possible. To do so would require semi-permanently installing cameras into the ceiling and walls of a multi-purpose operating room. To maximize the view of the environment while allowing users to fully visualize the procedure during filming, the 180-degree stereoscopic camera was placed against the wall of the surgical suite. If no activities were taking place in the operating room beyond the operating table, we would locate the camera closer to the procedure for a better view. This allowed users to be able to fully visualize all activities within the setting while also allowing our camera operators to relocate the equipment for use across different suites and settings.

As we move forward, we plan to acquire more Meta Quest 2 headsets as well as have the instructor bring the headsets from the library so that each team group can utilize them during the class to fully immerse themselves into the clinical procedures. We believe that this will allow for more accurate data collection with regard to the educational effectiveness of higher quality VR headsets. Moreover, further refinement and development of our interactive learning modules via a PC version made available for anyone with a computer should also enhance the pedagogical effectiveness of our virtual learning experiences. To this end, we plan to develop a game-like hospital environment where students can enter different surgical suites that will display VR medical procedures, interviews, simulated medical student training procedures (e.g., emergency room simulation-based training https://youtu.be/UnrQQs7hxdU or those presented in Singh et al. [42]), and other video content associated with the devices utilized or how the procedure is performed. This multi-format deployment of our educational content using 2D interview videos and 3D VR experiences made accessible across a range of devices (mobile, PC, and VR headsets) and platforms including YouTube and our course database, lends itself to affordable scalability to include any number of students, classes, and disciplines. It should prove to be effective in remote learning settings as well as being used as educational content in a traditional classroom setting. Though access to high-quality VR devices is currently cost-prohibitive for large-scale deployment, continued technological advancement will eventually drive down costs to an acceptable level for mass deployment.

The phenomenological effect of 3D VR technology allows us to psychologically and sensorially immerse the users in a virtual space to a greater degree than traditional 2D video content. As one of our students’ described it, VR “allows you to immerse yourself in the environment. I mean turning your head kind of puts you in the feeling that you are in the operating room. So I would definitely say that in terms of immersion, I would *definitely* prefer watching the videos on the Meta Quest over YouTube.” The embodiment granted by VR technology, allowing the user to participate in the virtual environment through a first-person perspective, increases the users’ felt sense of presence: of being psychologically and even physically present within the virtual environment. Given the highly subjective nature of the sensation of presence in a virtual space, adequate comprehension of the human user experience is paramount in further developing the effectiveness of virtual learning environments usage in undergraduate BME training. VR’s ability to increase the sense of presence and, at least in some cases, allow for better focus thanks to the lack of distractions while wearing an HMD shows promise in developing more effective virtual learning environments across a wide range of disciplines.

Because VR learning environments are in their nascent stage of development and practical application for undergraduate education, it remains unclear as to what functionalities and interactive elements will be the most crucial for student learning. Understanding the way in which students interact with virtual learning environments as well as what they want from such environments will be paramount. Such user feedback should help us determine the types of features that will be the most important in creating virtual learning environments that more effectively teach a concept, specific body of knowledge, and enhance the development of practical skills like clinical needs finding and screening. Further integration of digital technologies which track users' physiological data like eye tracking, pupil dilation, heart rate, heart rate variability, and brain activity will enable the development of learning mechanisms that adapt to each individual’s learning pace. Therefore, while development in functionality, visual fidelity, and user interactivity will be needed, we believe that the integration of VR learning environments will ultimately evolve to become more pedagogically effective than traditional 2D video content.

## Figures and Tables

**Fig. 8:**
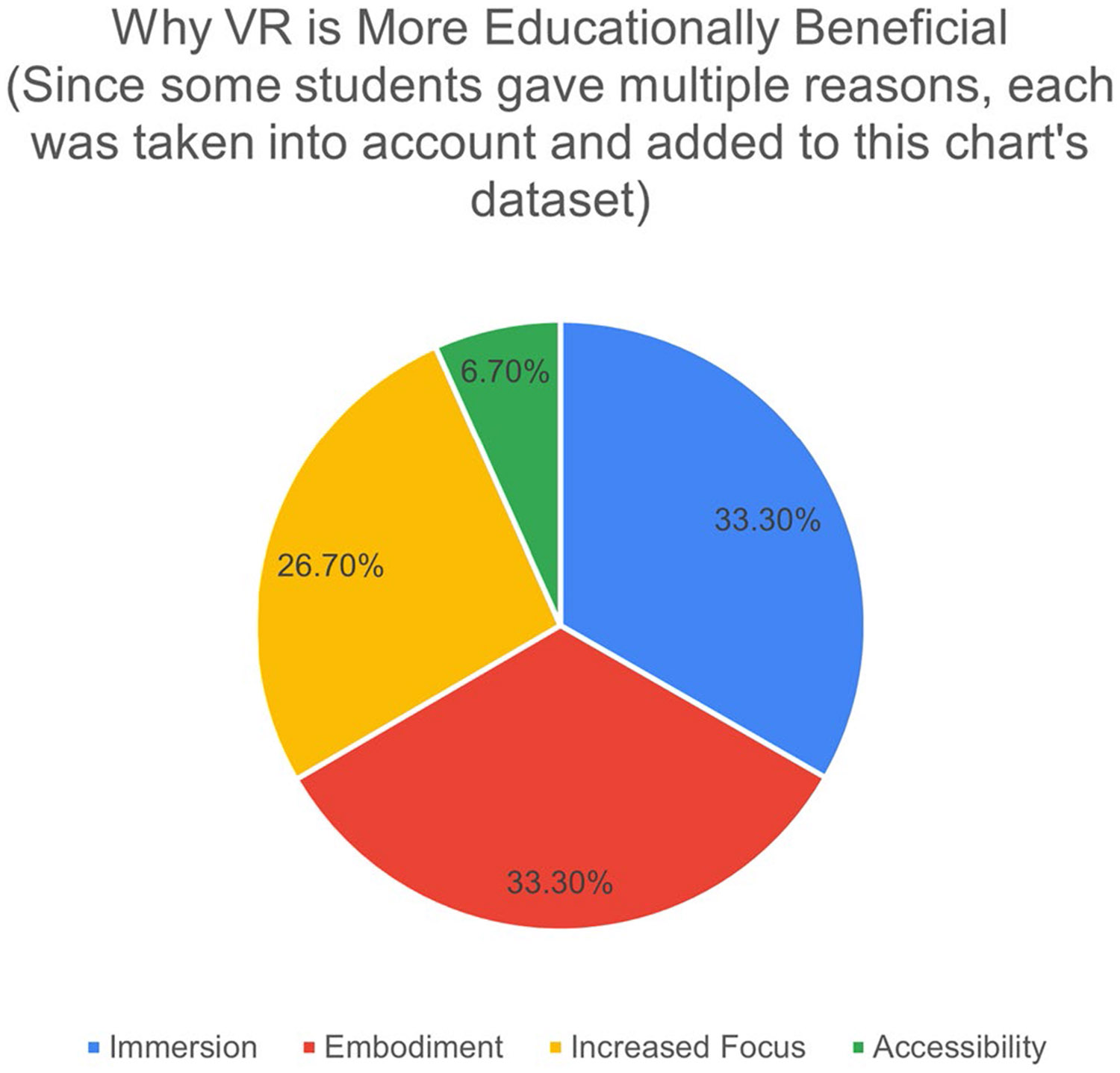
Percentages of the responses to the post-course survey question “Why VR is More Educationally Beneficial?” (No. Responses = 20 students)

**Fig. 9:**
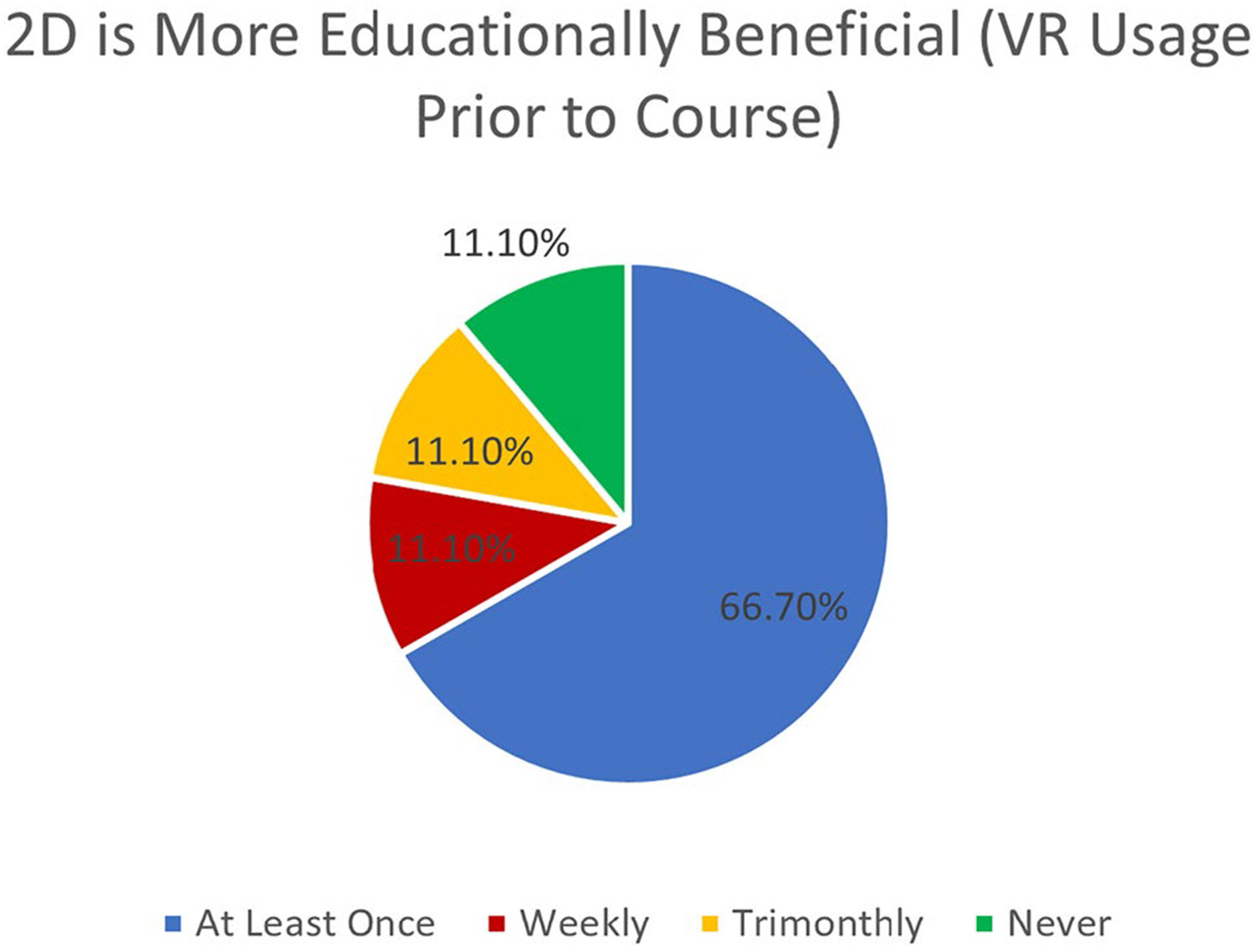
Percentages of respondents to “2D is more educationally beneficial” when asked about VR usage prior to the course (No. Responses = 20 students).

**Fig. 10:**
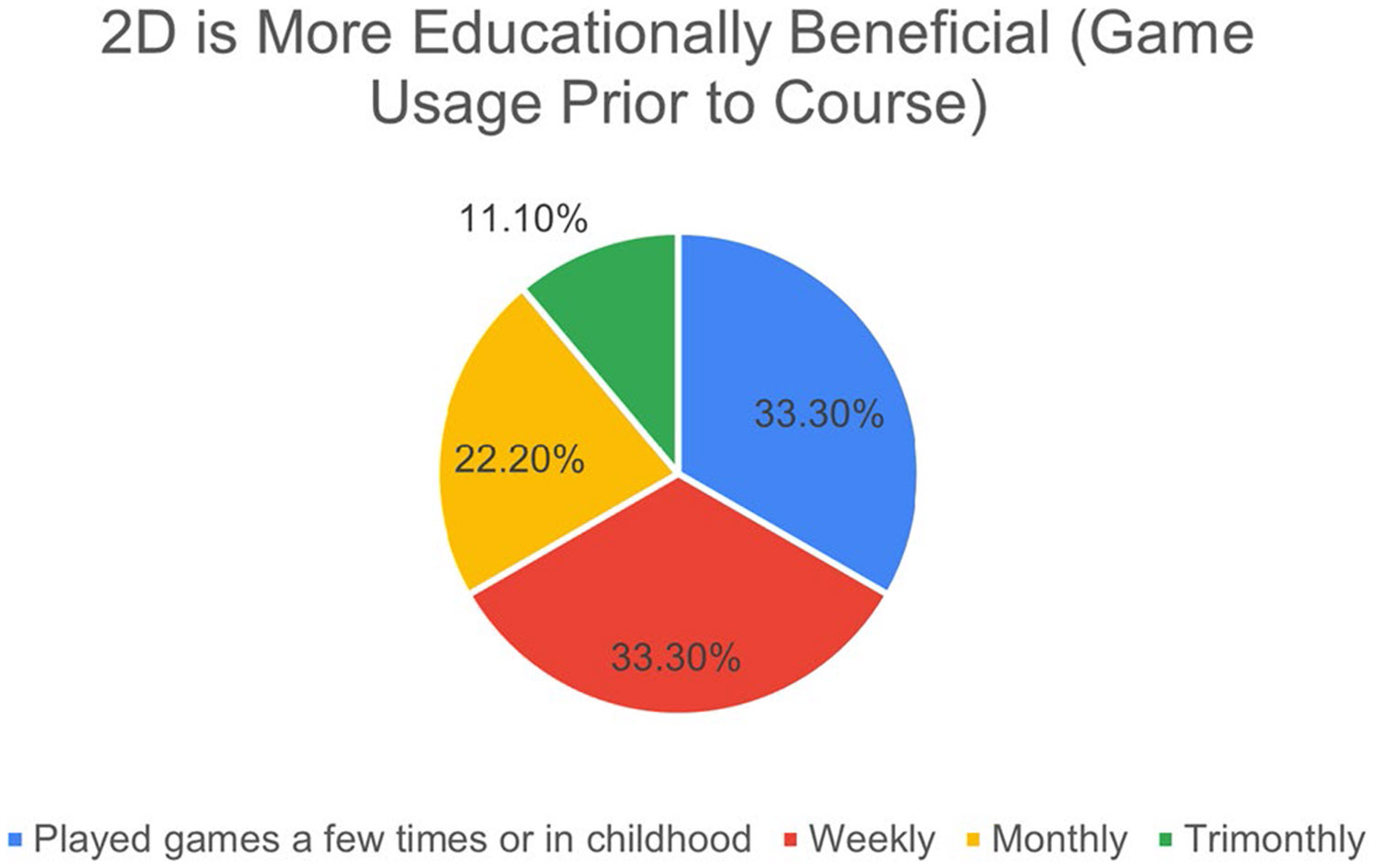
Percentages of respondents to “2D is more educationally beneficial” when asked about video game usage prior to the course (No. Responses = 20 students)

**Fig. 11:**
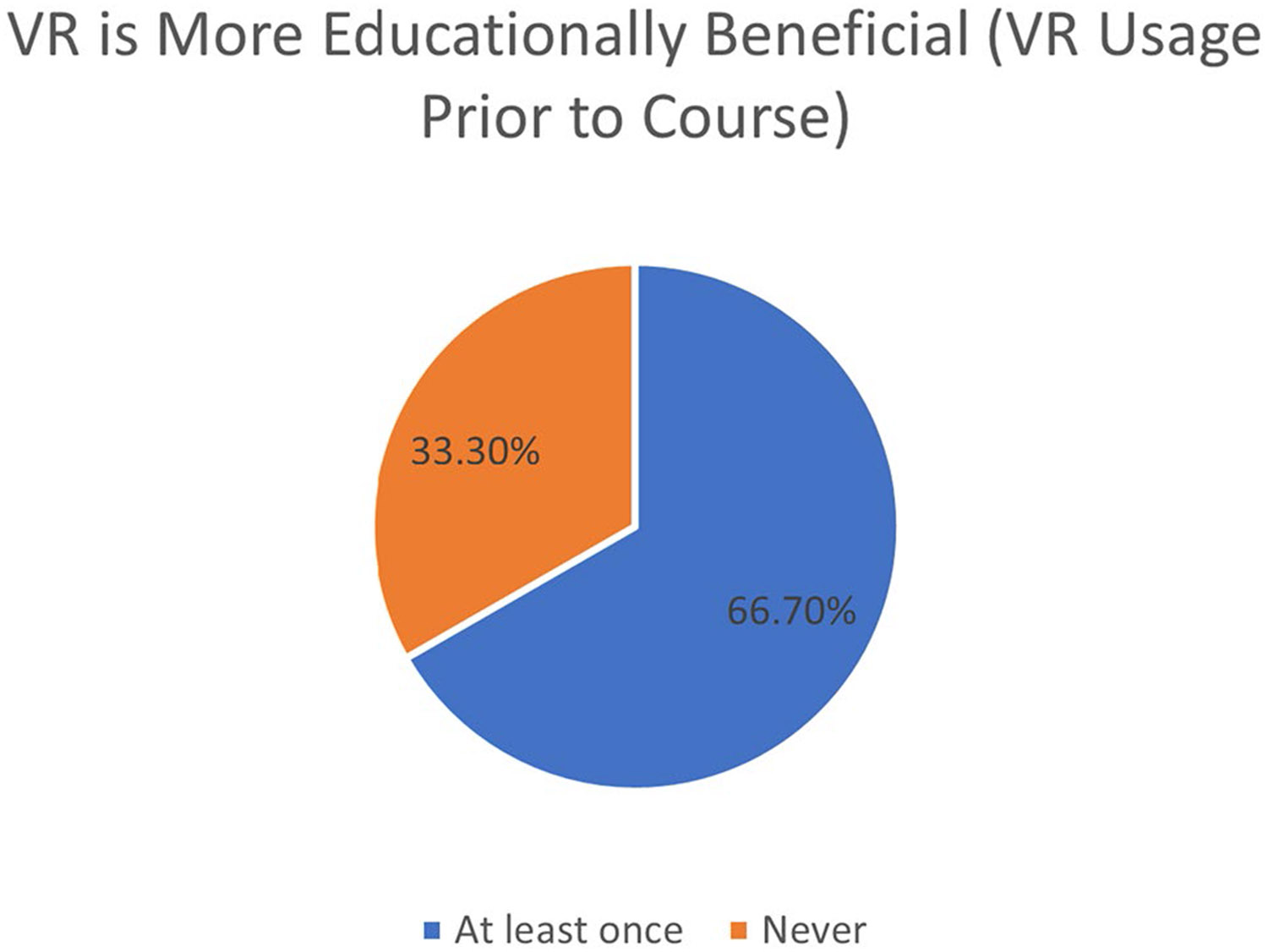
Percentages of respondents to “VR is more educationally beneficial” when asked about VR usage prior to the course (No. Responses = 20 students)

**Fig. 12:**
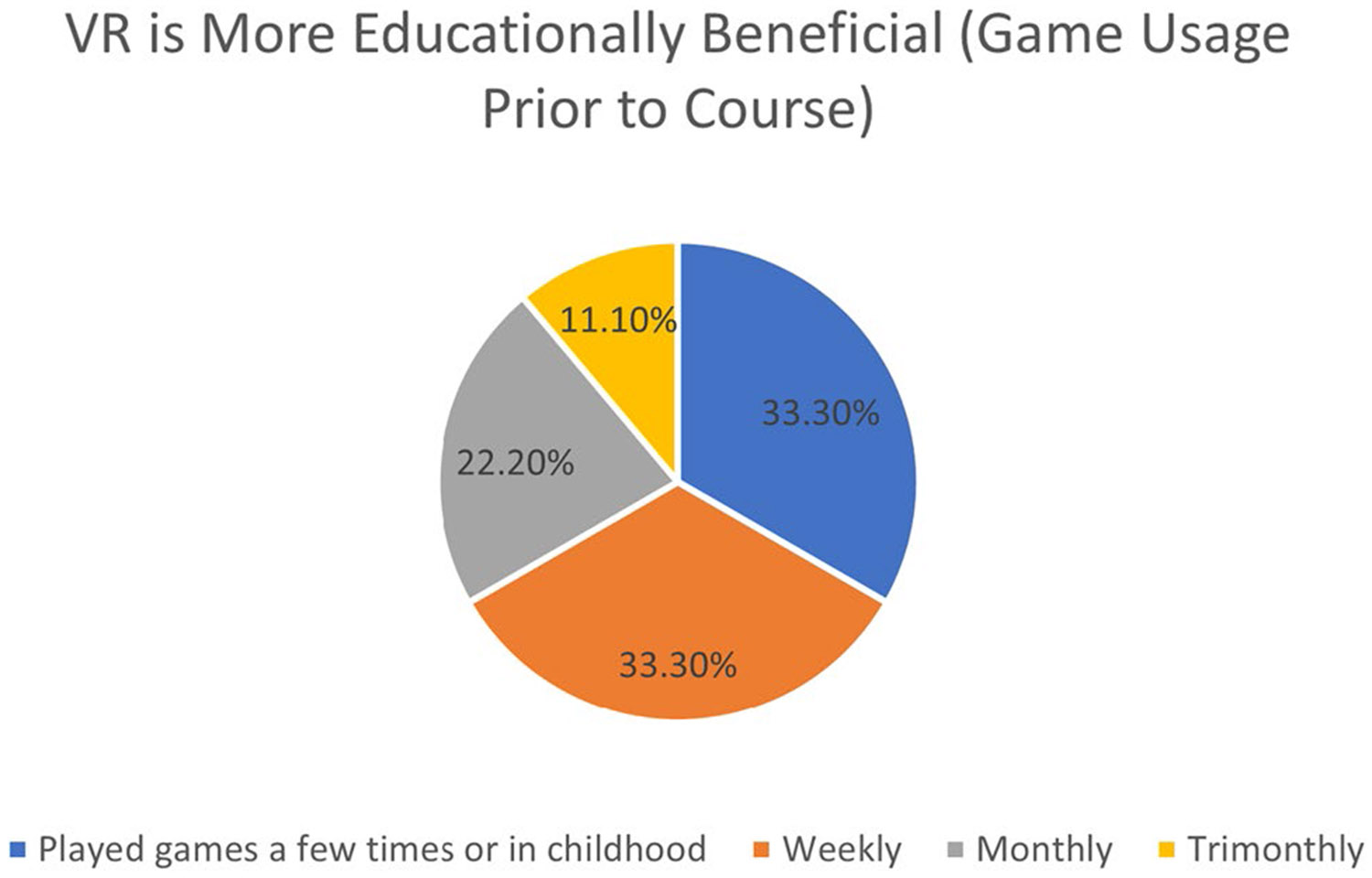
Percentages of respondents to “VR is more educationally beneficial” when asked about video game usage prior to the course (No. Responses = 20 students)

**Fig. 13:**
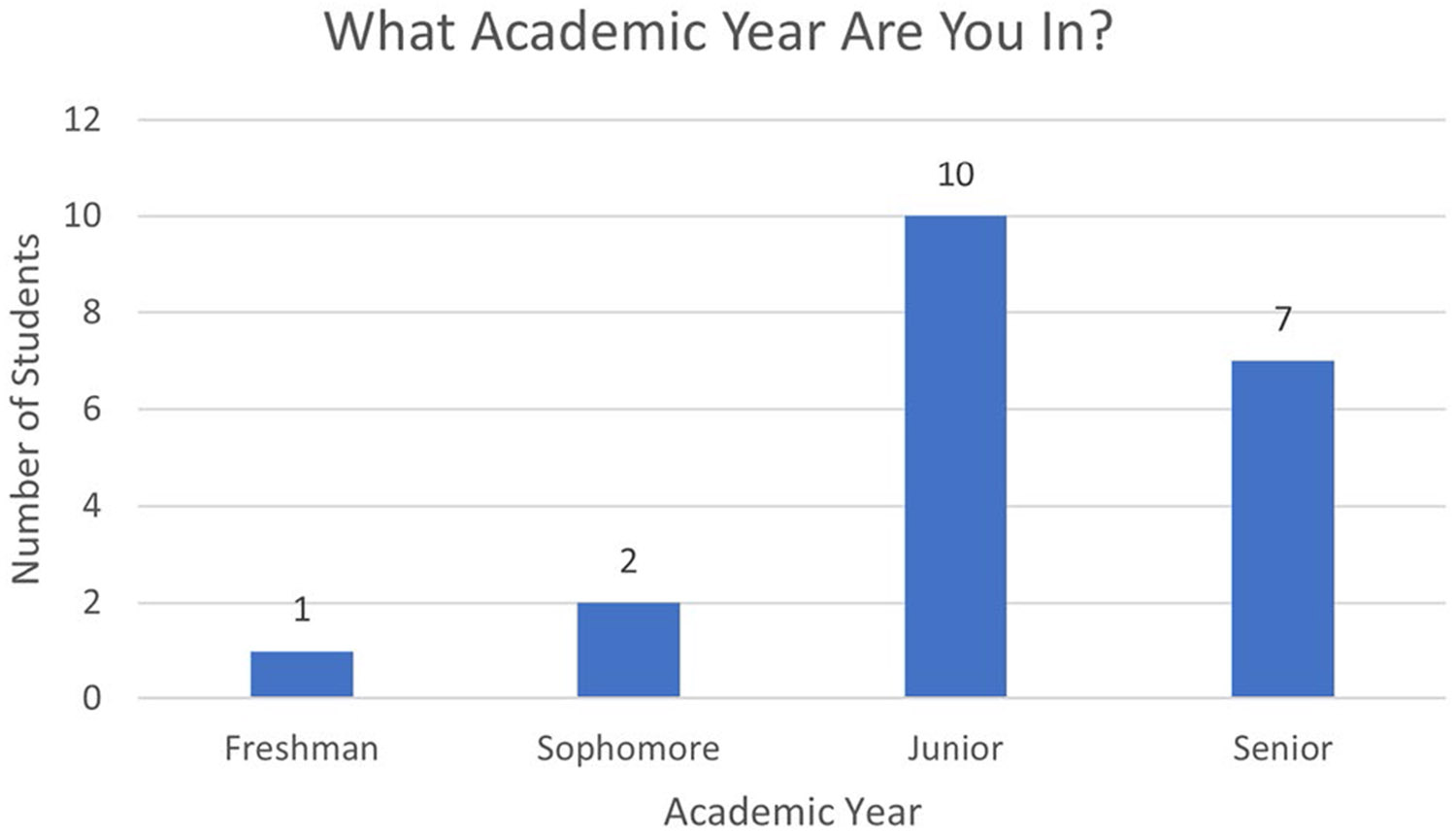
Responses to the survey question “What academic year are you in?”

**Fig. 14:**
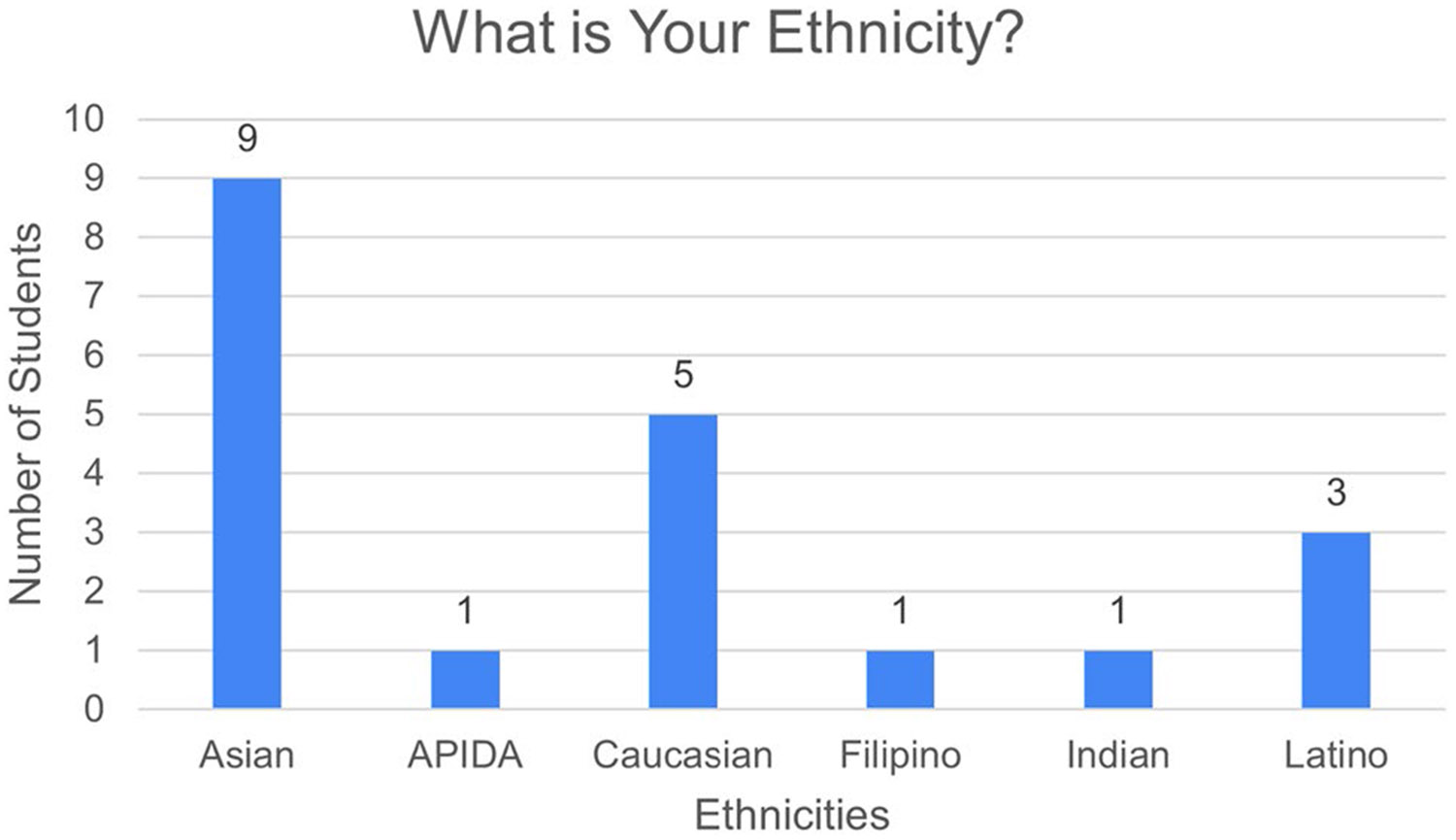
Responses to the survey question “What is your ethnicity?”

**Fig. 15:**
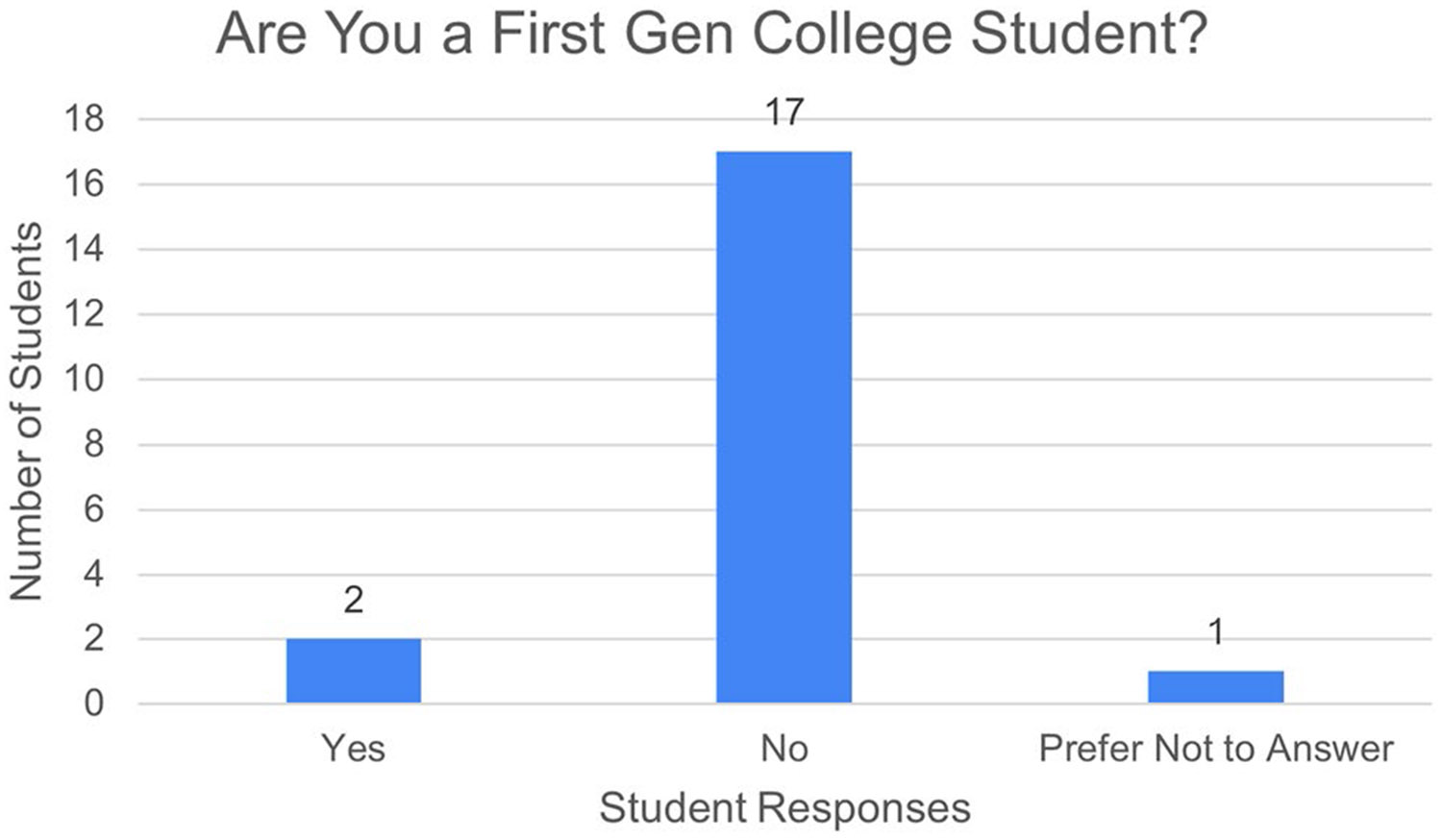
Responses to the survey question “Are you a first-generation college student?”

**Fig. 16:**
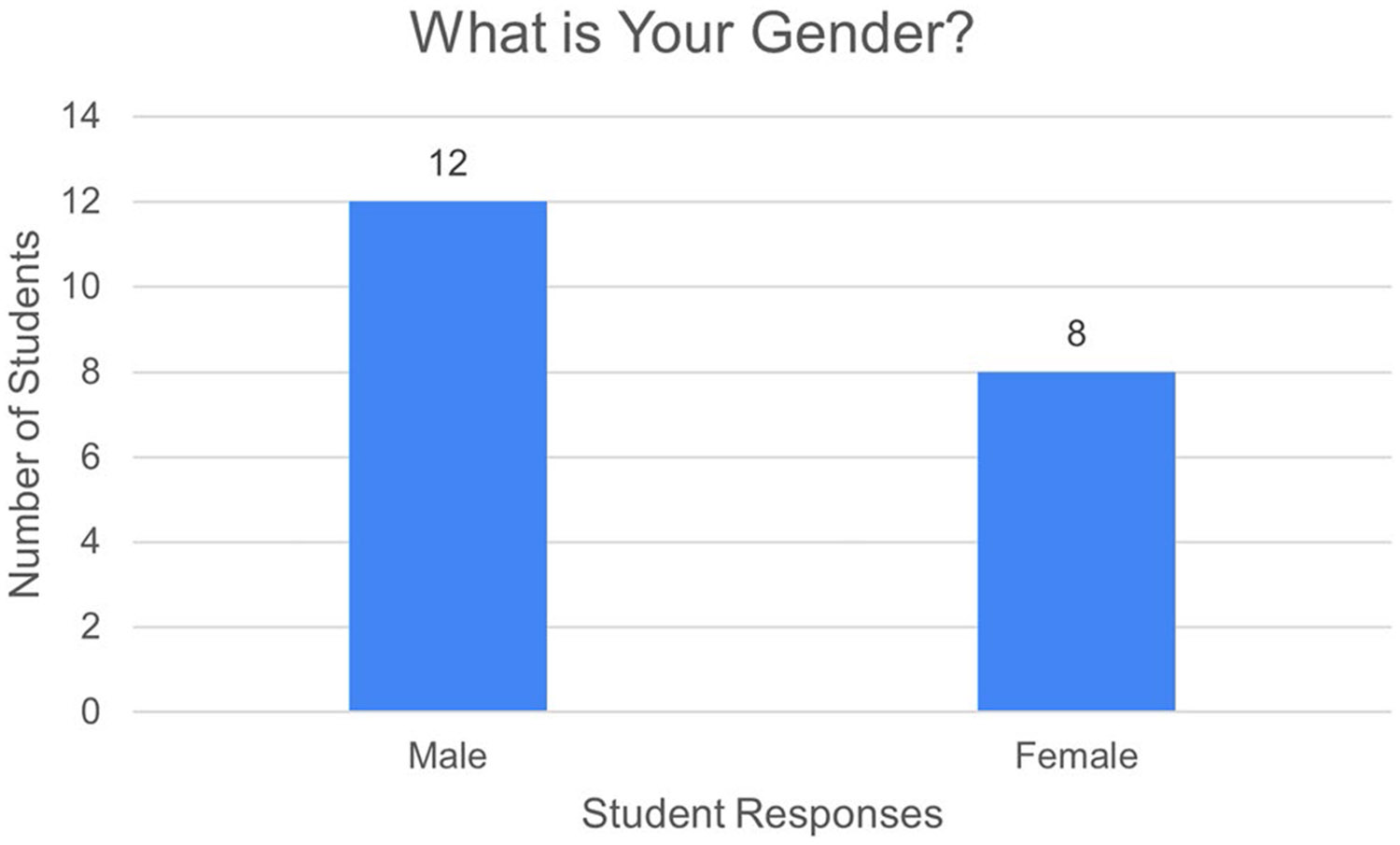
Responses to the survey question “What is your gender?” Note that zero students responded as “non-binary” or “prefer not to answer.”

## Data Availability

All data and course materials can be provided by emailing the author: kingce@uci.edu.

## Appendix A: Figures

See Figs. 8, 9, 10, 11, 12, 13, 14, 15, 16

## Appendix B: Surveys and Interviews

### B.1 Survey 1: Submitted Prior to Taking the Course

1. What do you consider your ethnicity to be?
2. What academic year are you in?
  - Freshman
  - Sophomore
  - Junior
  - Senior
3. What is your gender?
  - Male
  - Female
  - Non-binary
  - Prefer not to answer
4. Are you a first-generation college student?
  - Yes
  - No
  - Prefer not to answer
5. What is your familiarity with VR?
  - I have never used VR
  - I have used VR at least once
  - I use VR at least once every three months
  - I use VR at least once a week
6. What is your familiarity with video games in general (PC gaming, console gaming, mobile gaming)?
  - I have never played a video game
  - I have played video games a few times or only when I was a child
  - I play video games at least once every three months
  - I play video games at least once a week
7. How do you think VR might be useful for helping to develop clinical needs finding skills? Please list the aspects of VR that you think will be educationally beneficial vs. traditional methods such as videos.
8. What aspects of VR do you think might be an impedance to developing clinical needs finding skills? Please list any potential negatives you think VR will pose to your education.
9. Are you excited or interested in using VR for educational purposes? Please explain why or why not.

### B.2 Survey 2: Submitted After Taking the Course

1. Which experience did you find to be the most beneficial to your learning?
  - VR Version: Meta Quest 2
  - VR Version: Google Cardboard
  - PC Version
  - Traditional 2D Video Style (i.e., YouTube)
  - All were equally beneficial
2. Which experience did you find to be the least beneficial to your learning?
  - VR Version: Meta Quest 2
  - VR Version: Google Cardboard
  - PC Version
  - Traditional 2D Video Style (i.e., YouTube)
  - None were noticeable beneficial
3. Did the VR experiences elicit a greater degree of immersion, presence, and/or embodiment (the feeling that you are actually present in the operating room/clinical environment)?
  - Yes
  - No
  - All were equally immersive
  - None of them were immersive
4. Which VR version did you think was the most educationally beneficial and why (VR: Meta Quest 2 or VR: Google Cardboard)?
5. Which version did you find most effective and why (PC, VR: Meta Quest 2, VR: Google Cardboard, Traditional 2D Video Style [YouTube])? (Please try to key in on specific details of the experience that made it better than the other versions. For instance, perhaps the first- person perspective and interactive controls of the VR: Meta Quest 2 experience enhanced immersion or your engagement with the material leading to a better learning experience overall).
6. Which version did you find the least effective and why (PC, VR: Meta Quest 2, VR: Google Cardboard, Traditional 2D Video Style [YouTube])? (Please try to key in on specific details of the experience that made it worse than the other versions).
7. Please list any ideas you have or potential features for improving the VR: Meta Quest 2 and PC experiences. (For instance, having a multiplayer function that would allow groups to meet together in the virtual space or the ability to increase playback speed).
8. Please list any educational use cases where you think VR programs could be effectively used to improve learning and why you think it will be more effective than traditional in-class or online learning methods. For instance: a virtual space where you could meet up in groups for more effective remote teamwork. Or, a virtual learning environment where everyone meets using avatars for online classes rather than zoom lectures. Interactive virtual spaces designed to teach a specific concept or body of knowledge. Something akin to an interactive museum exhibits wherein you can engage with the material individually or as a group.

### B.3 Interview Questions: Conducted After Taking the Course

1. Did you find the VR experience to be more immersive and/or provide an increased sense of embodiment? Please describe what you liked and disliked and how the VR environment made you feel.
2. Do you think an increased sense of immersion and embodiment will be better for clinical immersion education? Why or why not? Please try to list specific elements to support your opinion.
3. What elements should be added or improved to make the VR versions more educationally beneficial?
4. What could be improved about the overall experience of our educational content and experiences in general?

## Appendix C: Sample of Student Devised Interview: Questions and Answers

Doctor Q and A.

**Q:** Can you talk about which type of hernia is more comment in patients of different genders?
  **A:** Groin hernia. Indirect , direct (usually men) Because testicle descent from chest down to groin.Leaves defect Femoral (usually women) Women have round filament that creats smaller defect
**Q:** Why no bleeding? Why don't I see a lot of bleeding like in a TV show?
  **A:** Not breaking the skin or big blood vessels.
    Not a lot of bleeding because we are not messing around with big blood vessels or the skin. Also, the bleeding that you see are probably even less since what you saw is magnified.
**Q:** Are the Meshes & sutures biodegradable? Why are we using different types of mesh? For example, the self-fixating ones and the larger ones that you used AbsorbaTack to staple.
  **A:** No(and) Type of mesh depends on type of hernia.(Gravity, weight, etc …)
**Q:** Won’t holes from surgery leave defacts for future hernia?
  **A:** No . (Because) Dialating troachar used.
    And the wound is small.
    For the trocars we used, they are less than or about 8mm. If the wound is above 8 mm we would need to suture it up.
**Q:** What is the liquid that you inject into the patient at the cut site before cutting?
  **A:** Those are local anesthesia for pain.
**Q:** Can you feel, or have any kind of pressure/ force feedback when controlling the robot?
  **A:** No.
**Q:** Do you think having feedback when you are controlling the robot can be a good thing that can be improved?
  **A:** It is not a big problem if we got used to it. It's good but it might not change much.

Question to doctor's assistant.

**Q:** Do you think a vacuum cart for cleaning up after the surgery would be helpful?
  **A:** Not really. We don’t have a lot of trash to clean up after.
